# A Trojan Horse for live-cell super-resolution microscopy

**DOI:** 10.1038/s41377-019-0238-7

**Published:** 2020-01-06

**Authors:** Gerti Beliu, Markus Sauer

**Affiliations:** Department of Biotechnology and Biophysics, Biocenter, University of Würzburg, Am Hubland, 97074 Würzburg, Germany

**Keywords:** Applied optics, Optical techniques

## Abstract

New peptide vehicles enable the efficient live-cell labeling of intracellular organelles with cell-impermeable fluorescent probes by simple coincubation, paving the way for refined multicolor super-resolution fluorescence imaging.

Presently, the most popular imaging modality in cell biology is fluorescence microscopy, due to its unique combination of molecule-specific labeling, minimal perturbation and three-dimensional (3D) imaging. In the last decade, super-resolution microscopy has evolved into a very powerful method for the fluorescence imaging of cells at subdiffractional resolution and structural investigations of cellular organelles^1,2^. Super-resolution microscopy methods can now provide a spatial resolution that is well below the diffraction limit of light microscopy, enabling invaluable insights into the spatial organization of proteins in biological samples. Fluorescent proteins are doubtless the labels of choice for live-cell super-resolution microscopy because they can be genetically fused to the protein of interest. Organic fluorophores such as rhodamine or carbocyanine dyes are substantially smaller and generally exhibit a higher fluorescence quantum yield and improved photostability but require a chemical labeling procedure. The posttranslational labeling of proteins in living cells can be achieved, for example, by using genetically encoded polypeptide tags in combination with organic fluorophore ligands^3,4^. However, specific and efficient labeling of intracellular organelles in living cells remains challenging mainly because of membrane impermeability and the nonspecific binding of organic fluorophores and ligands.

When selecting the best fluorescent probe for the specific labeling of biomolecules, there is always a trade-off between biological and photophysical properties. Different aspects must be considered, such as the quantum yield, photostability, spectral properties, and cell permeability. In terms of water solubility and polarity, hydrophilic fluorescent probes are known to show less nonspecific binding but lack membrane permeability, whereas hydrophobic probes exhibit higher membrane permeability but tend to bind nonspecifically to cellular compartments or organelles^5^. In the context of drug delivery and membrane translocation of cell-impermeable biomolecules, cell-penetrating peptides (CPPs), which are able to cross the membranes of mammalian cells, are a promising class of molecules. Since their discovery over 30 years ago^6,7^, there has been an increasing number of newly identified CPPs with potential applications in the life sciences and therapeutic treatment. Because of their relatively small sizes (typically <50 aa), CCPs can function as peptide vehicles (PVs) and are capable of delivering different macromolecules, including DNA, proteins or chemical probes, into living cells.

Recently, Zhang et al.^8^ introduced a straightforward and easy-to-implement strategy for the efficient membrane translocation of cell-impermeable organic fluorescent probes. Despite recent progress in the generation of unassisted cell-permeable fluorescent probes^9–13^, the majority of organic fluorophores are cell impermeable, which therefore limits their applicability to live-cell labeling of intracellular targets. Based on the work of Erazo-Oliveras et al.^14^, they investigated different disulfide-bonded dimers of short (12-amino acid long) peptides containing different peptide sequences and fluorophores and discovered that a positively charged peptide vehicle (PV-1) outperformed all other vehicles. Interestingly, the peptide dimer was modified covalently with two rhodamine B molecules, whose presence greatly improved the delivery efficiency of nonpermeable fluorescent probes. On the other hand, the rhodamine B dyes did not show substantial fluorescence due to the formation of a nonfluorescent “closed” lactone isomer in the charged peptide vehicle^8^. Hence, the two dyes do not interfere with the fluorescence of the delivered fluorescent probes, thus allowing for multicolor imaging applications.

By applying micromolar concentrations of PV-1 and different cell-impermeable fluorescent probes, the intracellular structures of live cells, including microtubules, actin, nuclei, lysosomes, mitochondria and the ER, could be efficiently and specifically labeled^8^. Metaphorically speaking, PV-1 functions like the historic “Trojan Horse” by smuggling uninvited guests through a locked door (Fig. 1). PV-1 enables delivery of up to three different fluorescent probes simultaneously into live cells for the specific labeling and imaging of various intracellular structures by multicolor structured illumination microscopy (SIM). Analogously to previously described compounds^14^, the presented protein vehicles utilize the ability to escape from endosomes, resulting in cytosolic release. In addition, the presented results indicate the involvement of a caveolae- and clathrin-independent endocytic pathway and thus support previous reports of micropinocytosis being the main mechanism involved in membrane translocation. In comparison to previously described peptide vehicles, PV-1 shows enhanced performance and enables the efficient delivery of organic fluorescent probes via simple coincubation. The results also indicate that optimized compound design, e.g., the insertion or substitution of fluorophores into peptide segments, is essential for efficient function. By varying the fluorophore and chemical linkage, cytosolic delivery efficiency can be modulated without further affecting the viability of the treated cells, suggesting new possibilities for the design, synthesis and application of future peptide vehicles.

**Fig. 1:**
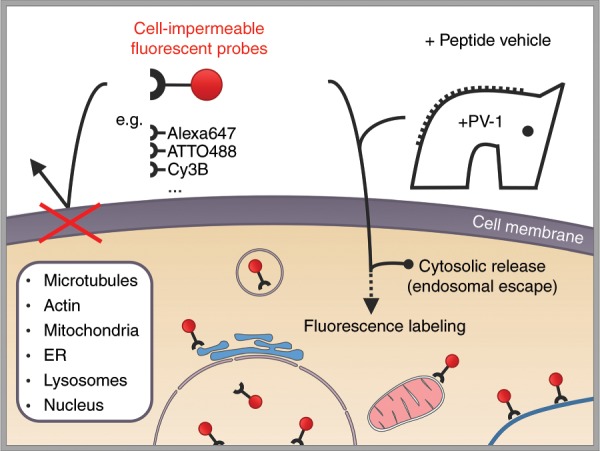
The Trojan Horse. Schematic of the intracellular delivery of cell-impermeable fluorescent probes via coincubation with the peptide vehicle PV-1. A positively charged dimeric peptide vehicle (PV-1) enables the membrane translocation of fluorescent probes into the cytosol and the specific labeling of intracellular structures, e.g., microtubules, actin, nuclei, lysosomes, mitochondria, and the ER.

The described peptide vehicle will find numerous applications in the fluorescence imaging of intracellular proteins in living cells, especially when demanding super-resolution microscopy methods are used. The possibility of using cell-impermeable fluorescent probes for the specific labeling of intracellular targets will greatly extend the palette of fluorescent probes for live-cell fluorescence imaging. In combination with unnatural amino acid incorporation by genetic code expansion and specific labeling with small bioorthogonal clickable fluorogenic tetrazine dyes^15^, the introduced peptide vehicles will facilitate the refined live-cell imaging of intracellular structures with high spatial and temporal resolution and as-of-yet unattained freedom in terms of fluorophore choice.
